# Molecular imaging for evaluation of synovitis associated with osteoarthritis: a narrative review

**DOI:** 10.1186/s13075-023-03258-6

**Published:** 2024-01-16

**Authors:** Kwanghoon Lee, Soheil Niku, Sonya J. Koo, Ernest Belezzuoli, Monica Guma

**Affiliations:** 1 Department of Medicine, University of California San Diego, La Jolla, CA USA; 2 Department of Medicine, Dongguk University Ilsan Hospital, Goyang, Korea; 3 Nuclear Medicine Service, Jennifer Moreno VA San Diego Healthcare System, San Diego, CA USA; 4 Department of Radiology, West Los Angeles VA Medical Center, Los Angeles, CA USA; 5 Department of Radiology, University of California San Diego, La Jolla, CA USA

**Keywords:** Osteoarthritis, Synovitis, Synovial inflammation, PET-CT

## Abstract

Recent evidence highlights the role of low-grade synovial inflammation in the progression of osteoarthritis (OA). Inflamed synovium of OA joints detected by imaging modalities are associated with subsequent progression of OA. In this sense, detecting and quantifying synovitis of OA by imaging modalities may be valuable in predicting OA progressors as well as in improving our understanding of OA progression. Of the several imaging modalities, molecular imaging such as positron emission tomography (PET) and single-photon emission computed tomography (SPECT) has an advantage of visualizing the cellular or subcellular events of the tissues. Depending on the radiotracers used, molecular imaging method can potentially detect and visualize various aspects of synovial inflammation. This narrative review summarizes the recent progresses of imaging modalities in assessing inflammation and OA synovitis and focuses on novel radiotracers. Recent studies about imaging modalities including ultrasonography (US), magnetic resonance imaging (MRI), and molecular imaging that were used to detect and quantify inflammation and OA synovitis are summarized. Novel radiotracers specifically targeting the components of inflammation have been developed. These tracers may show promise in detecting inflamed synovium of OA and help in expanding our understanding of OA progression.

## Background

Osteoarthritis (OA) is one of the most common musculoskeletal disorders affecting over 300 million people globally [1]. OA poses a substantial burden on the daily living of affected patients. Kiadaliri et al. showed that patients with knee OA had substantially more health care consultations, use of medications, and net disability days [2]. Due to the aging of populations, the annual incidence of OA has greatly increased with 102% increase in crude incidence rate between 1990 and 2017 [3], representing a significant burden to healthcare for societies worldwide.

Traditionally, OA has been considered as a disease of cartilage degeneration, but recent studies suggest a role of low-grade synovial inflammation in the progression of OA. Synovitis at baseline detected by magnetic resonance imaging (MRI) or ultrasound (US) is associated with radiographic progression of OA [4, 5]. Synovitis progression is associated with more cartilage damage [6] and mediates the association of obesity and knee OA radiographic progression [7]. Erosive hand OA and accelerated knee OA are also related to synovitis [8, 9].

Various cellular components comprise the low-grade synovitis of OA. Single-cell RNA sequencing (scRNA-seq) identified 12 different expression profiles in OA synovium including synovial sub-intimal fibroblasts, synovial intimal fibroblasts, HLA-DRA + cells, smooth muscle cells, endothelial cells, T cells, mast cells, and proliferating immune cells [10]. Macrophages, synovial fibroblast-like synoviocytes (FLS), neutrophils, and endothelial cells are the major players in the progression of OA [11].

In this regard, detecting and quantifying OA synovitis, as well as the specific cellular types, are valuable in advancing our understanding of the complex disease process of OA. Imaging tools such as US, MRI, and molecular imaging allow for visualization and quantification of synovitis associated with OA. Among these imaging modalities, molecular imaging allows for morphologic, cellular, and metabolic evaluation. Commercially available PET/SPECT radiotracers can be utilized in the novel context of OA. Specific radiotracers developed in research can be utilized to target specific immune cell types. In this narrative review, we briefly review the currently available imaging modalities to visualize synovitis and focus on recent advances in PET/SPECT imaging for evaluating synovitis in OA.

## Main text

### Imaging methods to assess synovitis

#### Ultrasound

Ultrasound (US) is an imaging modality that utilizes high-frequency sound waves in order to generate images of the body and can be used to evaluate for synovitis. Typical sonographic features of synovitis include a hypoechoic intra-articular structure that is neither compressible nor displaceable [12]. US can also differentiate synovitis from joint effusion. In addition, power Doppler US (PDUS) displays the strength of Doppler signal in color and may assess the vascularity of the synovium [13]. Power Doppler (PD) signal shows significant correlation with histologically assessed vascularity of large joints [13] but the correlation between PD signal and histologically assessed vascularity needs further study. US has been clinically useful when evaluating synovitis of rheumatoid arthritis (RA), in which early detection of synovitis is important. In the case of OA, knee joint effusion detected by US predicted subsequent joint replacement [14], and synovitis of hand OA detected by US predicted radiographic progression [15]. The degree of synovitis detected by PDUS is milder in OA than in RA [13], which may make detection of synovitis of OA with PDUS somewhat difficult. Novel imaging techniques such as superb microvascular imaging and contrast-enhanced US were developed to overcome this issue [16, 17], but more studies are needed to clarify their performances in detecting low-grade synovitis.

#### MRI

MRI allows for superior visualization of soft tissues including joint spaces, effusions, cartilage, synovium, ligaments, and tendons and is the current gold standard for imaging evaluation of synovitis. On conventional unenhanced MRI, synovitis is suggested by a thickened synovium that appears hypointense on T1-weighted images and hyperintense on T2-weighted images. Contrast-enhanced MRI using gadolinium-based contrast agents (GBCA) can significantly improve the accuracy of diagnosing synovitis by better demonstrating synovial thickening and also revealing prominent synovial enhancement due to hypervascularity of the inflamed synovium. However, contrast-enhanced MRI is more costly, more time consuming, and contraindicated in individuals who have renal dysfunction or GBCA allergies. Dynamic contrast-enhanced MRI (DCE-MRI) calculates the dynamic pharmacokinetics of GBCA enhancing the synovium and shows superior ability to assess synovitis compared to conventional MRI, but it is currently mainly used in the research setting.

#### Molecular imaging

PET and SPECT are molecular imaging techniques detecting gamma rays [18]. They have superior sensitivity than any other imaging modalities [19]. Unlike other imaging modalities, they can reveal the functional information in vivo depending on the radiotracers used. For example, ^18^F-FDG PET can detect lesions with high glucose metabolism such as tumor and infection. However, these modalities have limited spatial resolution and cannot provide precise anatomical information. Hence, they are often combined with CT or MRI as a single imaging system such as PET/CT, PET/MRI, or SPECT/CT. These hybrid imaging can provide both functional and anatomical status in vivo. In addition, PET can be obtained of the whole body whereas US and MRI assess only one or a few joints per session. Despite these advantages, molecular imaging, especially PET, is not utilized in the evaluation of OA yet, because we have limited evidence that they are useful in identifying OA phenotypes or in contributing to diagnosis, prognosis, and/or response to treatment. Table 1 summarizes the general advantages and disadvantages of the three imaging modalities mentioned above.

**Table 1:** General advantages and disadvantages of imaging modalities for synovitis

Imaging modality	Advantages	Disadvantages
Ultrasound	Low-cost, real-time imaging, relative ease	Operator dependent, small field of view, poor visualization of deeper joint structures
MRI	Gold standard for evaluation of joints anatomically and current gold standard for evaluation of synovitis	High cost, relatively small field of view, issues with claustrophobia, noisy, longer scan times
Molecular imaging (PET/SPECT)	Ability to detect specific cellular or molecular process in vivo depending on radiotracer utilized, ability to screen the whole body in one scan	High cost, limited spatial resolution, longer scan times

*MRI* magnetic resonance imaging, *PET* positron emission tomography computed tomography, *SPECT* single-photon emission computed tomography

### Radiotracers for evaluating inflammation

#### ^18^F-FDG

Various immune cells recruited to the sites of inflammation show high glucose metabolism and can be visualized by FDG-PET scans. After the introduction of commercially available PET/CT scanner [20], FDG-PET has been used widely in assessing inflammation in various infectious and inflammatory diseases. However, FDG-PET tracers do have some limitations. First, the specificity of FDG-PET for inflammation is relatively low. Most human cells consume glucose for the synthesis of ATP and hence physiologic FDG uptake is seen throughout the whole body. Malignant cells also consume glucose highly making discrimination between inflammation and malignancy difficult with stand-alone PET and no clinical information [21]. Second, specific measures to reduce the physiologic background FDG uptake such as fasting at least 4–6 h and avoiding voluntary movement are needed before performing PET scanning, which limits its use in critically ill patients [21]. Thirdly, certain drugs interfere with FDG uptake altering the sensitivity of FDG-PET scan. Insulin accelerates glucose uptake of cells and should be avoided before perming FDG-PET scans [22]. Metformin increases intestinal glucose uptake and may interfere with FDG-PET scan outcomes in patients taking metformin [23]. Antibiotics and glucocorticoid use for a long period also reduces the sensitivity of FDG-PET scans limiting its use in patients who are urgently in need of these medications [24, 25]. In this sense, PET/SPECT scans to detect inflammation using radiotracers other than 18F-FDG could be potentially useful overcoming these limitations of FDG-PET.

#### Novel radiotracers

Many novel radiotracers have been developed. Though many of these tracers were initially developed for oncologic purposes, several have shown promising results in demonstrating inflammation, both in human and animal studies (Table 2).

**Table 2:** Potential novel radiotracers for detecting inflammation

Targets of tracers		Tracers			Diseases/disease model studied	Studied for OA
Macrophage	TSPO	^11^C-(R)-PK11195 (first generation)		Clinical	Multiple sclerosis [26], atherosclerosis [27], large vessel vasculitis [28], rheumatoid arthritis [29]	
				Preclinical	Rat model of acute neuroinflammation [30]	
		^11^C-vinpocetine		Clinical	Multiple sclerosis [26]	
		^11^C-DPA-713		Preclinical	Rat model of acute neuroinflammation [30] Mice model of tuberculosis inflammation [31]	
		^18^F-DPA-714		Preclinical	Rat model of acute neuroinflammation [30] Rat model of abdominal aneurysm [32]	
		^11^C-DAC		Preclinical	Rat model of autoimmune encephalomyelitis [33]	
		^18^F-FEDAA1106		Clinical	Multiple sclerosis [34]	
		^18^F-FEDAC		Preclinical	Rat model of acute lung injury [35]	
		^18^F-PBR06		Clinical	Quantification of TSPO in normal human brains [36]	
		^11^C-SSR180575		Preclinical	Rat model of neuroinflammation [37]	
		^18^F-PBR111		Preclinical	Rat model of temporal lobe epilepsy [38]	
		^18^F-GE-180		Preclinical	Rat model of stroke [39]	
		^11^C PBR28		Clinical		Osteoarthritis [40]
	Surface receptor	Somatostatin receptor	^68^Ga-DOTATATE	Clinical	Atherosclerosis [41]	
			^64^Cu-DOTATATE	Clinical	Atherosclerosis [42, 43]	
			^68^Ga-DOTATOC	Clinical	Atherosclerosis [43]	
			^68^Ga-DOTANOC	Clinical	Cardiac sarcoidosis [44]	
		Macrophage mannose receptor	^99m^Tc-tilmanocept	Clinical	HIV related cardiovascular disease [45]	
			^18^F-FB-anti-MMR Ab	Preclinical	Mouse model of tumor [46]	
			^99^mTc-anti MMR nanobody	Preclinical	Mouse model of rheumatoid arthritis [47]	
			^111^In-Tilmanocept	Preclinical	Mouse model of atherosclerosis [48]	
		CXCR4	^68^Ga-Pentixafor	Preclinical	Rabbit model of atherosclerosis [49]	
		Folate receptors	^99m^Tc-EC20	Clinical	Rheumatoid arthritis [50]	Osteoarthritis [51]
			DTPA-folate	Preclinical		Rat model of osteoarthritis [52]
	Biologic pathways	Choline	^18^F-fluorocholine	Clinical	Atherosclerosis [53]	
		System XC	^18^F-FSPG	Preclinical	Mice model of multiple sclerosis [54]	
		Methionine	^11^C-Methionine	Clinical	Myocardial infarction [55]	
	Macrophage enzymes	iNOS:	^18^F-NOS	Clinical	Acute lung inflammation [56]	
		Cathepsins:	^68^Ga-BMV101	Preclinical	Mouse model of lung fibrosis, idiopathic pulmonary fibrosis [57]	
	Other macrophage specific tracers in preclinical studies	^64^Cu-Man-LIPs		Preclinical	Mouse model of lung cancer [58]	
		^64^Cu -Cinnamoyl-F-(D)L-F-(D)L-F (cFLFLF)			Mouse model of diabetes [59]	Rat model of osteoarthritis [60]
		^64^Cu-D-Ala1-peptied T-amide (DAPTA)			Mouse model of vascular injury [61]	
		^99m^Tc-NbV4m119 (CRIg-targeting nanobodies)			Mouse model of rheumatoid arthritis [62]	
		^99m^Tc-anti-Sn mAb (SER-4) (CD169 targeting)			Mouse model of allograft rejection [63]	
		Anti-Vsig4 Nbs (nanobodies targeting Vsig4)			Mouse model of hepatitis [64]	
		^99m^Tc-labelled anti-CD11b Ab			Mouse model of atherosclerosis [65]	
Fibroblast	FAP inhibitor	^68^Ga-FAPI		Clinical	Cancer [66], IgG4 related disease [67, 68], dilated cardiomyopathy [68]	
		^68^Ga-DOTA-FAPI-04		Clinical	Cancer [69]	Osteoarthritis [70]
Angiogenesis	VEGF/VEGFR pathway	^89^Zr-Df-bevacizumab		Clinical	Cancer [71–75]	
		^89^Zr-Df-ranibizumab		Preclinical	Mouse model of cancer [76]	
		^64^Cu-NOTA-RamAb		Preclinical	Mouse model of cancer [77]	
	α_v_β_3_-integrin	^18^F-FPRGD_2_		Preclinical	Cancer [78]	
				Clinical		Osteoarthritis [79]
		^68^Ga-PRDG2		Clinical		Osteoarthritis [80]

##### Radiotracers targeting macrophages

Macrophages are one of the most abundant cell types found in OA synovium and are reported to comprise 12–40% of the entire OA synovial cell population [10, 81, 82]. Activated macrophages have been regarded as a major player in the pathogenesis of OA as well as in other inflammatory diseases. Macrophages are classically categorized into M1 (proinflammatory) and M2 (anti-inflammatory) subtypes depending on their function, surface markers, and secreted molecules, though there are also macrophage populations that do not belong to either of these two groups. Single-cell RNA sequencing of OA synovium identified heterogeneous groups of cells, including M1 and M2 macrophages as well as unclassified macrophage subtypes [10]. Of note, macrophages are known to be highly plastic depending on the microenvironment they encounter. In this sense, redirecting macrophage polarity from proinflammatory to anti-inflammatory and joint-repairing subtypes might be a valuable treatment option, and clarifying and comparing macrophage subtypes of OA synovium with those of other conditions might be meaningful. Several macrophage-specific targets have been identified and radiotracers directed against these targets have been developed and studied in various inflammatory diseases.

1. *Translocator protein (TSPO) targeting tracers*: TSPO is expressed on the outer membrane of mitochondria. TSPO is expressed in both M1 and M2 macrophages, but some studies reported that it is more highly expressed in M2 subset [83, 84] TSPO targeting PET tracers were developed in the 1980s and have been extensively studied mainly in neuroinflammatory disorders. Microglial cells are activated in various neurodegenerative diseases and enhance the expression of TSPO on the outer membrane of mitochondria [85]. ^11^C-(R)-PK11195, the first-generation TSPO radiotracer, has shown promising results not only in neuroinflammatory disease but also in other inflammatory diseases including atherosclerosis, large vessel vasculitis, and rheumatoid arthritis [26–30]. However, ^11^C-(R)-PK11195 had some clinical limitations such as the relatively short half-life of ^11^C, nonspecific binding, high background blood-pool accumulation, and performance variation depending on genetic polymorphism (rs6971 SNP) in the TSPO gene. Newer TSPO targeting PET tracers have been subsequently developed to overcome these shortcomings and have shown better performance in terms of bioavailability, signal-to-noise ratio, nonspecific binding, and non-displaceable binding potential compared to first-generation TSPO targeting tracers [26, 30–39, 86].
2. *Surface receptor targeting PET tracers*: Several macrophage surface receptors have been suggested as potential targets for imaging inflammation. Somatostatin receptor (SSTR) is a G-protein-coupled receptor found in various tissues and immune cells. SSTR2 has been shown to be highly expressed on M1 macrophages [41]. SSTR targeting PET scans have been used mainly in patients with neuroendocrine tumors, but the utility of these PET tracers has also been demonstrated in cardiovascular inflammation [42–44, 87]. Uptake of ^68^Ga-DOTATATE, ^64^Cu-DOTATATE, and ^68^Ga-DOTATOC correlated with cardiovascular risk scores [43, 87], and with vulnerable plaques and CD163 expression [42]. ^68^Ga-DOTANOC PET-CT showed superior diagnostic accuracy in cardiac sarcoidosis compared to conventional ^18^F-FDG-PET scans [44]. Macrophage mannose receptor (MMR/CD206) has also been suggested as a potential target for imaging macrophages. CD206 is used as a marker for detecting M2 macrophages [88] ^99m^Tc-tilmanocept uptake, a SPECT radiotracer targeting MMR, correlated with non-calcified aortic plaque volume in patients with HIV [45]. CXCR4 is a chemokine receptor found in immune cells and upregulated in various oncological conditions. CXCR4 + macrophages were reported to exhibit M2 phenotype [89]. CXCR4-specific PET tracer, ^68^Ga-Pentixafor, was initially developed for cancer imaging, but also correlated with histologic regions immunostained for CXCR4 and macrophage-specific marker in animal atherosclerosis model [49]. Folate receptors are also potential targets for imaging inflammation. Folate is a major source for the synthesis of DNA and RNA. There are three subtypes of folate receptors (FRα, FRβ, and FRγ) and FRβ is exclusively expressed in activated macrophage especially the M2 subtype [90]. ^99m^Tc-EC20, a FRβ-specific SPECT radiotracer, detected more active joints than those detected by physical examination by rheumatologists [50].
3. *Tracers targeting biologic pathways*: Some PET tracers target biologic pathways of macrophage activation. Choline is a precursor of phosphatidylcholine, which is a major source of cell membrane. Choline is reported to be taken up highly by active tumor cells and macrophages [91]. Choline transporter is reported to be expressed in both M1 and M2 macrophages [19]. ^18^F-fluorocholine (FCH) uptakes were reported to correlate with plaque areas immunostained with CD68. ^18^F-FCH uptakes were higher in symptomatic plaques than in asymptomatic plaques [53]. System XC is a membrane bound transporter involved in cysteine/glutamate uptake and is reported to be upregulated in activated M1 macrophages [92]. ^18^F-FSPG, a ^18^F labeled glutamate derivative PET tracer, is specifically taken up by system XC and has been shown to be superior to ^18^F-FDG in detecting early inflammatory changes of CNS in a mouse encephalitis model [54]. ^11^C-Methionine (^11^C-MET) PET tracer was shown to be taken up by active macrophages in tumor lesions and thus has been used in imaging neuro-oncological disease. It has also been shown to have a role in imaging inflammatory disease including atherosclerosis [55]. Uptake assay showed that ^11^C-MET uptake was sevenfold higher in M1 macrophages compared to M2 subtypes [93].
4. *Tracers targeting macrophage enzymes*: Some tracers target certain enzymes upregulated in macrophages. Inducible nitric oxide synthase (iNOS) produces nitric oxide, which is highly upregulated in M1 macrophages. The uptake of ^18^F-NOS, a NOS-specific PET radiotracer, was found to increase by 30% from baseline after induction of human lung inflammation [56]. Cathepsins are cysteine proteases that are upregulated in macrophages. They are found both in M1 and M2 macrophages with a predominance in M1 macrophages [94–96]. The uptake of ^68^Ga-BMV101, a cathepsin-specific PET radiotracer, was significantly higher in fibrotic lung lesions of patients with idiopathic pulmonary fibrosis than in those of control patients [57].
5. *Macrophage targeting radiotracers in preclinical studies*: Many radiotracers are being developed to improve the specificity of currently available tracers for targeting macrophages. ^64^Cu-Man-LIPs, ^99m^Tc-anti MMR, ^18^F-FB-anti-MMR, ^68^Ga-NOTA-MSA, and ^111^In-Tilmanocept were reported in disease models of tumor [46, 58], rheumatoid arthritis [47], and atherosclerosis [48]. In addition, peptide and antibody-based tracers have also been developed. Formyl peptide receptor 1 [Cinnamoyl-F-(D)L-F-(D)L-F (cFLFLF)] and CCR5 antagonists [D-Ala1-peptied T-amide (DAPTA)] were radiolabeled and tested for macrophage targeting imaging studies [59–61]. Antibody-based tracers that target macrophage surface receptors, including CD 163, CD11b, CD169, and CRIg, have also been developed and tested in disease models of atherosclerosis, host versus graft disease, and rheumatoid arthritis [59, 62–65]. Some nanoparticle tracers such as ^89^Zr-oxalate and ^64^Cu-TNP are also taken up by macrophages [97, 98].

##### Radiotracers targeting fibroblasts

Pathologic fibroblasts, more specifically activated OA fibroblast-like synoviocytes (OA FLS), produce proinflammatory cytokines, chemokines, and proteolytic enzymes which contribute to cartilage degradation and OA progression [99]. In this sense, PET tracers targeting activated fibroblasts may have a role in imaging inflammation of OA.

##### Small molecule inhibitors

Fibroblast activation protein (FAP), a membrane-bound protein, is highly expressed in cancer-related fibroblasts in most epithelial tumors and has been investigated as a potential therapeutic target in oncology [100]. FAP inhibitors (FAPIs) coupled with chelators specifically bind to FAP with complete internalization [101–103]. ^68^Ga-FAPI PET-CT was shown to have better contrast and higher tumor uptake than ^18^F-FDG in 6 patients with different tumors [66]. ^68^Ga-DOTA-FAPI-04 PET-CT was shown to have a higher detection rate in malignant tumors than conventional ^18^F-FDG PET-CT [69]. Increased FAPI PET tracer uptakes were also seen in other non-malignant conditions including IgG4-related disease [67, 68] and heart diseases [104].

##### Radiotracers targeting angiogenesis

Endothelial cells also play a role in the pathogenesis of OA [105]. Angiogenesis occurs throughout the established OA synovium and seems to be related to the persistence of inflammation [106]. The serum and synovial fluid levels of endothelial-derived vascular endothelial growth factor (VEGF) correlate with the WOMAC score, radiographic severity, presence of osteophytes, and synovitis grade assessed by power Doppler signal [107]. Radiccthelial cells might have a utility in OA, but most of the endothelial cell targeted tracers were investigated in cancer patients.

1. *PET tracers targeting VEGF/VEGFR pathway*: Bevacizumab is a monoclonal antibody directed against VEGF-A. ^89^Zr-Df-bevacizumab has been clinically investigated in patients with various cancers [71–75]. ^89^Zr-Df-bevacizumab detected primary breast cancer [72] and showed a value in visualizing treatment response after receiving everolimus in patients with metastatic renal cell carcinoma [73]. Ranibizumab is a mAb Fab derivative of bevacizumab with a higher affinity for VEGF-A than bevacizumab. ^89^Zr-Df-ranibizumab was able to show serial changes of angiogenic features in ovarian tumor models after treatment with the kinase inhibitor sunitinib [76]. Ramucirumab is directed against VEGFR-2, and ^64^Cu-NOTA-RamAb, a ramucirumab-based PET tracer, was able to visualize VEGFR-2 expression in mice tumor models [77].
2. *PET tracers targeting α*_*v*_*β*_*3*_*-integrin*: Integrins are heterodimeric transmembrane glycoproteins having an important role in cell–cell- and cell–matrix-interactions. α_v_β_3_-integrin is highly expressed in tumor cells and activated endothelial cells facilitating metastases and angiogenesis [78]. Arg-Gly-Asp (RGD)-based PET tracers were mainly used for detecting α_v_β_3_-integrins. [^18^F]FPPRGD2 PET scans were compared with ^18^F-FDG-PET scans in detecting primary lesions and metastatic lesions in 35 patients with breast cancer [108]. [^18^F]FPPRGD2 PET showed a higher overall sensitivity and specificity compared to ^18^F-FDG-PET scans in detecting tumor lesions. ^68^Ga-PRDG2 PET imaging uses cyclic arginine-glycine-aspartic acid (RGD) peptide, which specifically binds to α_v_β_3_-integrin that has a pivotal role in angiogenesis.

### Radiotracers studied for evaluating synovitis of OA

#### Conventional radiotracers

##### 18F-FDG

**Fig. 1:**
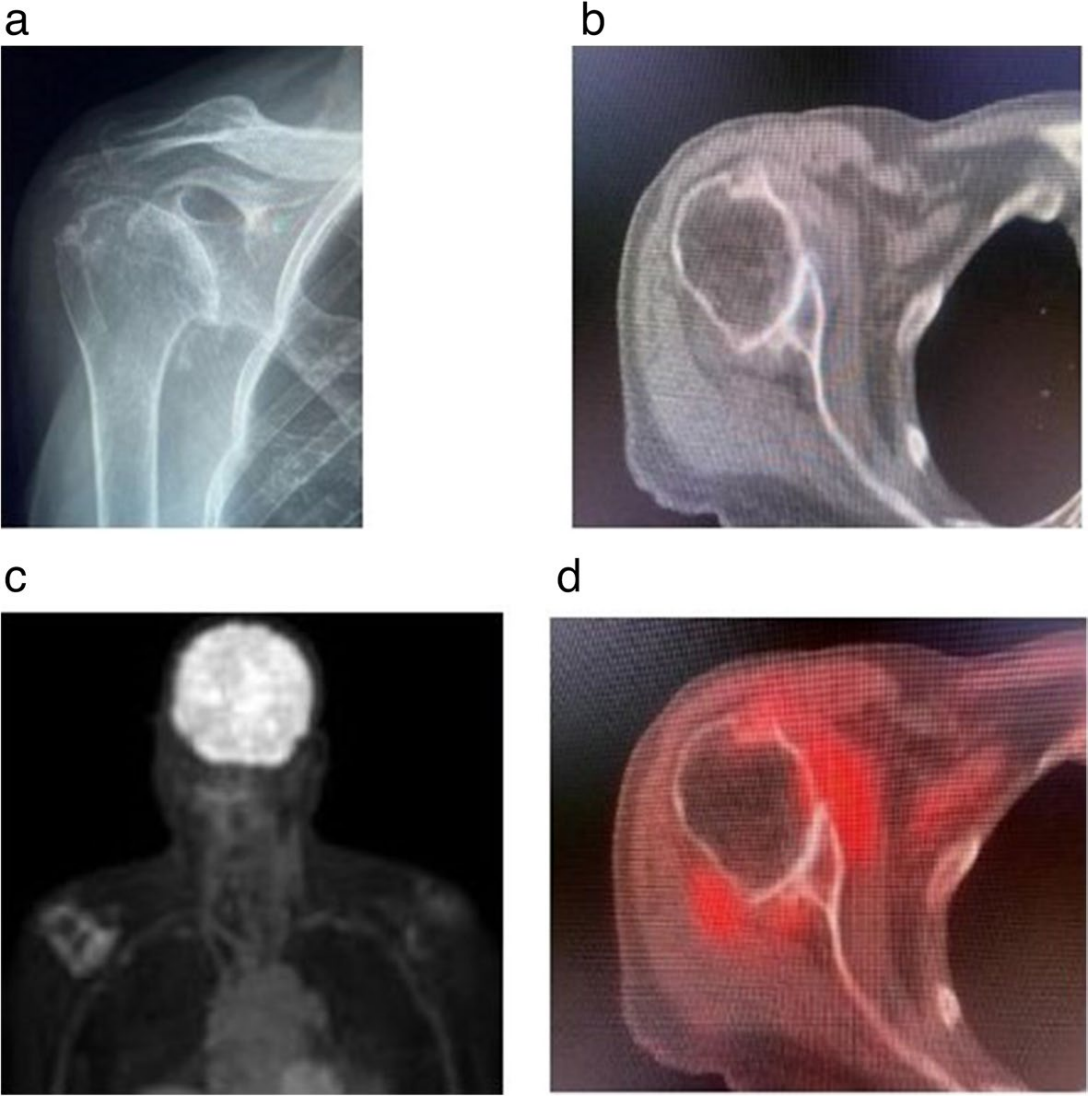
^18^F-FDG uptakes around right shoulder with osteoarthritis. **a** Frontal radiograph of the right shoulder shows glenohumeral joint OA with joint space narrowing and osteophyte formation. **b** Axial CT image at the level of the glenohumeral joints shows severe right glenohumeral joint space narrowing and osteophytosis, compatible with advanced OA. **c** MIP image from ^18^F-FDG PET-CT scan shows pathologic radiopharmaceutical accumulation around the right glenohumeral joint. **d** Fused axial and coronal PET-CT images show hypermetabolic activity along the periphery of the right glenohumeral joint in the expected location of the synovium and joint capsule, compatible with synovitis associated with OA

**Fig. 2:**
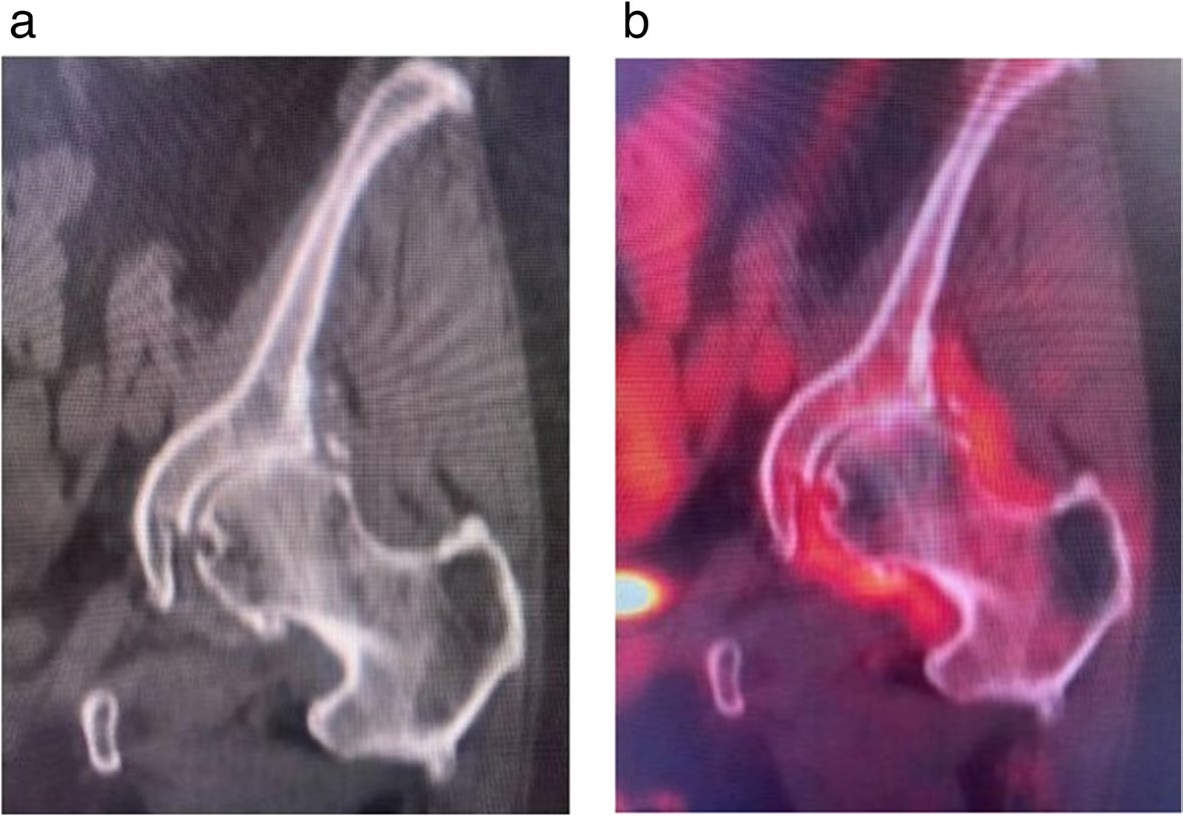
^18^F-FDG uptakes around left hip with osteoarthritis. **a** Coronal CT image of the left hip demonstrates joint space narrowing along the superior aspect of the left hip joint, compatible with mild-to-moderate OA. **b** Fused coronal ^18^F-FDG PET-CT image of the left hip shows hypermetabolic activity along the periphery of the left hip joint in the expected location of the synovium and joint capsule, compatible with synovitis associated with OA

**Fig. 3:**
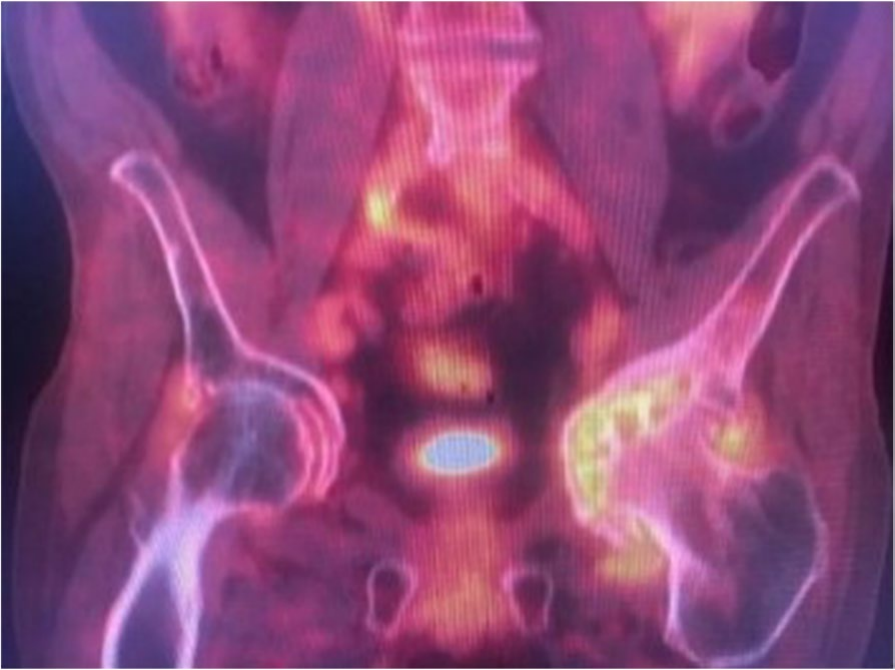
^18^F-FDG uptakes around the hip joints with osteoarthritis. Fused coronal ^18^F-FDG PET-CT image of the pelvis demonstrates severe left hip OA with advanced joint space narrowing, severe subchondral cyst formation particularly within the left femoral head, subchondral sclerosis, and acetabular remodeling (acetabular protrusion). In addition, extensive hypermetabolic activity is seen within the left femoral head suggestive of osteitis as well as along the periphery of the left hip joint suggestive of synovitis

Since ^18^F-FDG PET-CT can be utilized to evaluate inflammation, it can be helpful in revealing joints affected with OA, which also exhibit superimposed synovitis. The CT portion of the exam can reveal anatomic changes of OA, including joint space narrowing, subchondral sclerosis, and subchondral cyst formation. The PET portion of the exam may reveal areas of inflammation, including synovitis and osteitis. Figures 1, 2, and 3 reveal three separate cases of joints affected by OA which also exhibit synovitis. These cases show that ^18^F-FDG PET-CT can be valuable in identifying patients who have OA with superimposed overt synovitis, a finding that may have prognostic implications.

Nakamura et al. evaluated ^18^F-FDG PET-CT performed in 15 OA patients and compared the ^18^F-FDG uptakes with those of the asymptomatic controls [17]. They found that ^18^F-FDG uptake of OA patients were higher than controls, which was found mainly in the periarticular spaces, including intercondylar notch, periosteophytic lesions, and bone marrow. A preliminary study by Parsons et al. prospectively performed ^18^F-FDG PET-CT in 97 patients who were asked to complete knee pain questionnaires [109]. A total of 18 painful knees were identified and ^18^F-FDG uptake in these knees were compared with those in asymptomatic knees as control. The ^18^F-FDG uptake in painful knees were significantly higher than those of the control knees, suggesting that the magnitude of ^18^F-FDG uptake is directly related with knee pain. Nguyen et al. prospectively followed 65 patients with malignancy undergoing routine follow-up ^18^F-FDG PET-CT with acquisition of baseline values including Western Ontario and McMaster Universities Osteoarthritis Index (WOMAC) questionnaire and radiographs, and followed the patients for 5 years to determine the progression of OA [110]. ^18^F-FDG uptake was correlated with OA symptoms (WOMAC score) and radiographic stage (Kellgren-Lawrence score) and was independently associated with progression of OA in follow-up. Kogan et al. assessed the agreement between ^18^F-FDG PET-CT and MRI in 22 patients with knee pain or injury [111]. ^18^F-FDG uptake in regions with signs of OA (bone marrow lesions, osteophytes, and sclerosis) were significantly higher than in regions without degenerative changes on MRI. ^18^F-FDG uptake values in cartilage regions with grade 0 were significantly lower than in cartilage regions with grade 1–2 or 3–4, suggesting the potential role of this hybrid imaging in detecting metabolic and anatomical abnormalities of early OA. Similarly, Hong et al. found an association between ^18^F-FDG uptake and radiographic evidence of OA and symptoms of OA [112].

##### 18F-NaF

^18^F-NaF may be useful in the evaluation of bone metabolism. Menendez et al. demonstrated in a canine OA model that ^18^F-NaF uptake increases in the knees with anterior cruciate ligament transection (ACLT) [113]. Interestingly, the ^18^F-NaF uptakes increased in unaffected joints as well after ACLT. This suggests that ^18^F-NaF may potentially show altered bone metabolism early in the disease course of OA. Savic et al. performed ^18^F-NaF PET/MRI scans in 16 patients with early OA and found that increased cartilage T_1ρ_, which indicates degenerative changes, correlated with ^18^F-NaF uptake in adjoining subchondral bone, but with reduced ^18^F-NaF uptake in non-adjoining bones, highlighting the complex biomechanical and biochemical process in early OA [114]. Jena et al. also showed examples of ^18^F-NaF PET/MRI of OA patients in various stages, demonstrating increased ^18^F-NaF uptake in morphologically normal regions on MRI [115]. These findings suggest a role for ^18^F-NaF in the evaluation of early OA.

##### Radiotracers targeting macrophages

A PET scan using TSPO targeting radiotracer, [^11^C] PBR28 was assessed for its specificity to TSPO in a patient with OA, where it was found to bind specifically to lesions rich in TSPO in a patient with OA [40]. Folate receptor (FR) targeting radiotracers have also detected macrophage activation in experimental OA using diethylenetriaminepentaacetic acid (DTPA)–folate [52]. In addition, Kraus et al. performed SPECT/CT scans using ^99m^Tc-EC20, a FRβ targeting radiotracer in 25 patients with symptomatic knee OA, and showed that activated macrophages correlated with knee pain severity as well as the radiographic severity [51].

##### Radiotracers targeting angiogenesis

^68^Ga-PRDG2 PET scan uses cyclic arginine-glycine-aspartic acid (RGD) peptide, which specifically binds to α_v_β_3_-integrin that has a pivotal role in angiogenesis. Zhu et al. assessed the ^68^Ga-PRDG2 uptake in RA patients with OA patients as control. While the ^68^Ga-PRDG2 uptake was diffusely upregulated in the joints of RA patients, the ^68^Ga-PRDG2 uptakes were confined to specific regions in the joints of OA patients [80].

##### Radiotracers targeting fibroblast

Fenerciooglu et al. reported a case with uveal malignant melanoma and both knee OA, who underwent both FDG-PET/CT and ^68^Ga-FAPI4 PET/CT for cancer restaging. Both modalities detected OA changes of both knees, but ^68^Ga-FAPI4 PET/CT showed a higher affinity to the joints compared to FDG-PET/CT [70]. Withofs et al. evaluated ^18^F-FPRGD2 PET/CT scans taken from 62 cancer patients and searched for musculoskeletal lesions in these scans. ^18^F-FPRGD2 PET/CT detected more musculoskeletal lesions and showed higher target to background ratios compared to FDG-PET scans [79].

##### Clinical utility of cell-specific radiotracers in OA

The clinical application of cell-specific radiotracers in OA is an area that currently lacks substantial data. The current research focus revolves around determining whether each PET tracer will contribute to the diagnosis, prognosis, and/or in identifying OA phenotypes. Nevertheless, radiotracers targeting macrophages (via folate receptor) and fibroblasts appear promising. Notably, ^99m^Tc-EC20 demonstrated the ability to provide direct in vivo evidence linking macrophage activation to both knee OA symptoms and radiographic severity [51]. These specialized radiotracers hold potential for shedding light on the intricate cellular-level pathogenesis of OA in a non-invasive manner. Furthermore, their utility extends to assessing the response of novel treatments that specifically could target OA-related cell types. Additionally, these radiotracers may facilitate the identification of a subset of OA patients who could benefit from particular drugs, given the recognition of multiple inflammatory pathways as potent treatment targets for OA. Finally, exploring the distinctions in molecular imaging features between inflammatory arthritis and OA could uncover specific molecular imaging patterns unique to OA.

##### Future directions

Additional research is essential to fully understand the practical value of cell-specific radiotracers in the context of OA. Exploring the distinctive aspects of molecular imaging between inflammatory arthritis and OA could unveil unique molecular imaging patterns specific to OA. Recent advancements in imaging technology have led to the development of next-generation total-body PET scanners. These scanners offer significant improvements, including reduced dosage, faster acquisition times, and the ability for quantitative measurement of tracer uptake through dynamic image acquisition and kinetic modeling [116]. These advancements hold promise for the effective utilization of molecular imaging in OA. Among the radiotracers mentioned in this context, only 68 Ga-FAPI-04 has undergone studies for total-body PET imaging. Research employing kinetic modeling with total-body imaging of 68 Ga-FAPI-04 demonstrated superior quantification and enhanced lesion contrast compared to semiquantitative uptake measurements [117]. An additional consideration in evaluating candidate radiotracers for use in OA is the potential impact of radiometabolites on distorting the uptake pattern of the parent radiotracers. For instance, there have been observations regarding 18F-FDG, suggesting its intracellular entrapment as 18F-FDG-6P, which was previously thought to be non-metabolizable. However, recent evidence contradicts this, suggesting further metabolism beyond 18F-FDG-6P [118]. Addressing these nuances regarding radiotracer behavior and metabolism is crucial in determining the suitability and reliability of these imaging agents for effective application in the field of OA.

## Conclusion

Synovitis has been highlighted as having a role in the progression of OA. Detecting and measuring synovitis of OA with currently available as well as novel radiotracers will likely prove valuable in advancing our understanding of the progression of OA. PET-CT utilizing ^18^F-FDG is the current method that allows for visualization of inflammation and synovitis associated with OA. Several novel radiotracers, including those targeting specific cellular components of OA synovitis, may also show promise in the future and have been summarized in this review. Further study of these novel radiotracers in the context of OA synovitis is required.

## Data Availability

Not applicable.

## Abbreviations

OA: Osteoarthritis
PET: Positron emission tomography
US: Ultrasonography
MRI: Magnetic resonance imaging
PET-CT: Positron emission tomography-computed tomography
SPECT: Single-photon emission computed tomography
FLS: Fibroblast like synoviocyte
PDUS: Power Doppler ultrasonography
PD: Power Doppler
RA: Rheumatoid arthritis
GBCA: Gadolinium-based contrast agent
DCE-MRI: Dynamic contrast-enhanced MRI
FDG: Fluorodeoxy glucose
CT: Computed tomography
WOMAC: Western Ontario and McMaster Universities Osteoarthritis Index
TSPO: Translocator protein
SNP: Single-nucleotide polymorphism
SSTR: Somatostatin receptor
MMR: Macrophage se receptor
FCH: ^18^F-fluorocholine
FAP: Fibroblast activation protein
FAPI: Fibroblast activation protein inhibitor
VEGF: Vascular endothelial growth factor
ACLT: Anterior cruciate ligament transection
